# Supplementary material for: From Bench to Greenhouse: The Comparative Nano-Bio System Effects of Green-Synthesized TiO2-NPs and Plant-Growth-Promoting Microorganisms in Capsicum annuum
Source: Plants (Basel). 2025 Dec 2;14(23):3672. doi: 10.3390/plants14233672 (PMC12693930; doi:10.3390/plants14233672)
Supplement: Supplementary file 1 [file plants-14-03672-s001.zip › plants-3975597-supplementary.pdf]

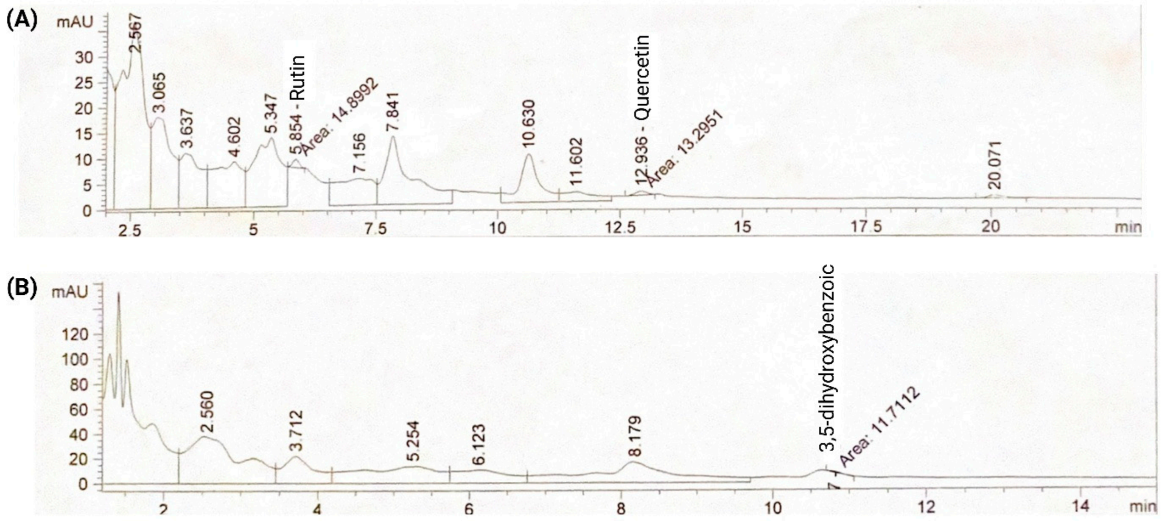

Figure S1. HPLC analysis of the isopropanol extract from blueberry.

Table S1. HPLC analysis of the methanol extract from blueberry. The listed compounds are according to available reports mentioned in [42].

Retention time (min)	Compound	Reference
9.859	Catechin	
10.327	Caffeic acid	
11.907	Procyanidine B2	
12.198	Chlorogenic acid	
12.585	Delphinidin-3-glucoside	
14.368	Delphinidin-3-arabinoside	[42]
14.819	4-hydroxycinnamic acid	
15.086	Petunidin-3-glucoside	
16.510	Peoinidin-3-glucoside	
17.307	Malvidin-3-glucoside	

Table S2. Structural properties of I- and M-TiO_2 utilized for the PLS analysis.

	**Chemical (Previous work, [34])**	**Green synthesis (present work)**		
	**Molten salt method**	**I-TiO_2**	**M-TiO_2**	**Units**
a	3.781	3.778	3.771	A
c	9.499	9.495	9.497	A
BG	3.02	2.98	3.01	eV
PS	41	12.91	12.42	nm

BET	7.38	41.65	46.51	m2/g
ZP	-50.06	-33	-50	mV
DLS	515	133	146	nm

Table S3. VIP for Nanoparticle Properties.

Physical properties	VIP	Interpretation
**DLS** (Hydrodinamic diameter)	**0.85**	High influence on biological responses
**PS** (particle size)	**0.77**	Moderate-high influence, key to growth/biochemical parameters
BG (bandgap)	0.004	Minimum contribution
BET (surface area)	0.004	Minimum contribution
ZP (Zeta potential)	0.002	Minimum contribution
